# Predictors of Participation in Mammography Screening among Non-Hispanic Black, Non-Hispanic White, and Hispanic Women

**DOI:** 10.3389/fpubh.2016.00188

**Published:** 2016-09-06

**Authors:** Cathy L. Melvin, Melanie S. Jefferson, LaShanta J. Rice, Kathleen B. Cartmell, Chanita Hughes Halbert

**Affiliations:** ^1^Cancer Control, Hollings Cancer Center, Medical University of South Carolina, Charleston, SC, USA; ^2^Department of Public Health Sciences, Medical University of South Carolina, Charleston, SC, USA; ^3^Department of Psychiatry and Behavioral Sciences, Medical University of South Carolina, Charleston, SC, USA; ^4^College of Nursing, Medical University of South Carolina, Charleston, SC, USA; ^5^Health Equity and Rural Outreach Innovation Center, Ralph H. Johnson Veterans Affairs Hospital, Charleston, SC, USA

**Keywords:** risk factors, cancer, mammography, screening, health knowledge, attitudes, practice

## Abstract

**Introduction:**

Many factors influence women’s decisions to participate in guideline-recommended screening mammography. We evaluated the influence of women’s socioeconomic characteristics, health-care access, and cultural and psychological health-care preferences on timely mammography screening participation.

**Materials and methods:**

A random digit dial survey of United States non-Hispanic Black, non-Hispanic White, and Hispanic women aged 40–75, from January to August 2009, determined self-reported time of most recent mammogram. Screening rates were assessed based on receipt of a screening mammogram within the prior 12 months, the interval recommended at the time by the American Cancer Society.

**Results:**

Thirty-nine percent of women reported not having a mammogram within the last 12 months. The odds of not having had a screening mammography were higher for non-Hispanic White women than for non-Hispanic Black (OR = 2.16, 95% CI = 0.26, 0.82, *p* = 0.009) or Hispanic (OR = 4.17, 95% CI = 0.12, 0.48, *p* = 0.01) women. Lack of health insurance (OR = 3.22, 95% CI = 1.54, 6.73, *p* = 0.002) and lack of usual source of medical care (OR = 3.37, 95% CI = 1.43, 7.94, *p* = 0.01) were associated with not being screened as were lower self-efficacy to obtain screening (OR = 2.43, 95% CI = 1.26, 4.73, *p* = 0.01) and greater levels of religiosity and spirituality (OR = 1.42, 95% CI = 1.00, 2.00, *p* = 0.05). Neither perceived risk nor present temporal orientation was significant.

**Discussion:**

Odds of not having a mammogram increased if women were uninsured, without medical care, non-Hispanic White, older in age, not confident in their ability to obtain screening, or held passive or external religious/spiritual values. Results are encouraging given racial disparities in health-care participation and suggest that efforts to increase screening among minority women may be working.

## Introduction

Since the introduction of mammography and the availability of more effective treatments, more than 60% of breast cancers are diagnosed at localized stages, resulting in a greater likelihood of higher 5-year survival rates (1). Further gains in 5-year survival may depend on increasing participation in mammography screening, improving our understanding of the role of genetics and of genetic screening, and developing more effective screening technology and treatment. Women and their health-care providers may find it difficult to share in informed decision-making about mammography screening given ongoing differences in approaches to assessing individual risk as defined by breast density and/or family history and in recommendations by various guideline developers regarding the appropriate age to begin screening, the periodicity of screening, and the value of different screening modalities (2–4). Without informed decision-making, women may modify their mammography screening behavior, risk being over-screened or under-screened based on current screening recommendations, and/or be less likely to maximize screening benefit. Both the ACS and the USPSTF suggest that decisions about mammography screening should be made on an individual basis taking into account patient context, including patient values regarding specific benefits and harms and preferences regarding breast cancer screening (5, 6).

Efforts to increase screening rates, reduce disparities in screening, and stimulate shared decision-making between women and their health-care providers about mammography screening require an understanding of motivators influencing women’s individual decisions to participate in screening. Prior work suggests that motivators include women’s awareness of the need for screening and of screening guidelines (7, 8), systems barriers to their utilization of screening services, and multidimensional personal preferences including psychological variables such as perceived risk of developing cancer as well as self-efficacy and culturally based values related to religion and spirituality, interpersonal relationships, autonomy, and temporal orientation (e.g., the extent to which individuals are concerned about the immediate or future consequences of behavioral options) (9, 10).

Our study aimed to explore the role of socioeconomic characteristics, health-care access, and cultural and psychological health-care preferences for cancer prevention and control on the prevalence of annual mammography screening in a national sample of racially diverse women in the United States (US).

## Materials and Methods

A national, random digit dial survey was conducted by a professional survey firm, Abt SBRI, between January and August 2009 prior to the release of the USPSTF recommendations in November of 2009. Eligible participants were individuals who were at least 18 years of age and self-identified as belonging to one of the following racial and ethnic groups: Hispanic Black, non-Hispanic White, or Hispanic. The American Association for Public Opinion Research (AAPOR) response rate for the survey was 47% resulting in a sample of 2133 US women (see Table 1). This population for this study consisted of a subset of 550 women aged 40–75 who self-identified as Hispanic Black (*n* = 210), non-Hispanic White (*n* = 181), or Hispanic (*n* = 159) and as having no personal history of any cancer. Study data were weighted using population targets for education, age, and gender from the March 2009 Current Population Survey for each racial group. Additional study methodology details are reported elsewhere (11).

**Table 1:** **Sample characteristics (*n* = 550 females)**.

Variable	Level	*n* (%) unweighted frequencies	*n* (%) weighted frequencies
**Socioeconomic factors**			
Race	Non-Hispanic Black	210 (38%)	60 (13%)
	Non-Hispanic White	181 (33%)	353 (76%)
	Hispanic	159 (29%)	53 (11%)
Marital status	Married	289 (53%)	273 (59%)
	Not married	261 (47%)	193 (41%)
Education level	>High school	319 (58%)	252 (54%)
	≤High school	231 (62%)	214 (46%)
Employment status	Employed	330 (60%)	269 (42%)
	Not employed	220 (40%)	196 (58%)
Income level	>$50,000	209 (38%)	196 (42%)
	≤$50,000	341 (62%)	269 (58%)
Age in years	Mean, SD	53.4, 9.3	54.7, 9.5
	Range	40–75	40–75
**Health-care access factors**			
Health insurance	Yes	471 (86%)	425 (91%)
	No	79 (14%)	41 (9%)
Usual source of medical care	Yes	514 (93%)	436 (94%)
	No	36 (7%)	29 (6%)
**Psychological factors**			
Self-efficacy for breast cancer screening	Completely confident	256 (47%)	220 (47%)
	Very confident	190 (34%)	162 (35%)
	Somewhat confident	78 (14%)	74 (16%)
	A little confident	16 (3%)	5 (1%)
	Not at all confident	10 (2%)	5 (1%)
Perceived risk of breast cancer	Very low	255 (46%)	173 (37%)
	Somewhat low	140 (25%)	131 (28%)
	Moderate	119 (22%)	121 (26%)
	Somewhat high	22 (4%)	23 (5%)
	Very high	14 (3%)	15 (3%)
**Cultural factors**			
Present temporal orientation	Strongly disagree	11 (2%)	6 (1%)
	Disagree	114 (21%)	93 (20%)
	Neutral	32 (6%)	35 (8%)
	Agree	267 (48%)	226 (48%)
	Strongly agree	126 (23%)	106 (23%)
Interpersonal relationships	Mean, SD	24.4, 5.1	24.2, 5.1
	Range	7–35	7–35
Religiosity	Mean, SD	21.4, 5.6	20.3, 6.0
	Range	6–30	6–30
Autonomy	Mean, SD	22.2, 4.2	22.2, 3.8
	Range	7–30	7–30

*“n” means sample size; “SD” means standard deviation*.

We created a three-level variable for race (non-Hispanic Black, non-Hispanic White, and Hispanic). Other study measures were obtained by self-report and included socioeconomic characteristics (e.g., race and marital status as reported in Table 1), health-care access variables (e.g., health insurance status and usual source of medical care as reported in Table 1), and cultural and psychological data on health-care preferences. Health-care preferences were measured in terms of beliefs related to perceived risk of developing breast cancer, self-efficacy about breast cancer screening, and cultural values about cancer prevention and control. Perceived risk and self-efficacy were evaluated using items from the Health Information National Trends Survey (HINTS) that asked women to estimate the likelihood of developing breast cancer compared to other women their age (1 = very low, 2 = somewhat low, 3 = moderate, 4 = somewhat high, and 5 = very high) and how confident they were in terms of obtaining breast cancer screening (1 = completely confident, 2 = very confident, 3 = somewhat confident, 4 = a little confident, and 5 = not at all confident), respectively (12). We re-coded responses to each of these items into conceptually meaningful categories used in previous research (11, 13). For perceived risk, these categories were at risk (somewhat or very high) and not at risk (very or somewhat low, moderate, and do not know), and the categories for self-efficacy were confident (completely or very confident) and not confident (somewhat, a little, or not at all confident).

Culturally based preferences were measured in terms of values related to religiosity, interpersonal relationships, temporal orientation, and autonomy. We used the Multi-Dimensional Cultural Values Assessment Tool (MCVAT) to evaluate values for cancer prevention and control (11). The MCVAT is a 19-item Likert-style tool to evaluate cultural values related to cancer prevention and control (CPC). The MCVAT was developed through qualitative research with African-American, White, and Hispanic adults who were asked to identify their values related to CPC. The MCVAT includes three subscales that evaluate religiosity or religious and spiritual values (e.g., it is important for me to pray before making a decisions about cancer screening), collectivist or interpersonal relationship values (e.g., I should talk to my family members about whether or not I should have cancer screening tests, it is important to me that my family supports my decisions about cancer screening), and autonomy or individualistic values (e.g., it is important for me to learn on my own about which cancer screening tests are needed) for CPC. The MCVAT subscales have acceptable internal consistency, with Cronbach’s alpha for the religiosity, interpersonal relationships, and autonomy subscales of 0.92, 0.79, and 0.73, respectively (11).

Temporal orientation was assessed by asking respondents how much they agreed or disagreed with the following statement: “it is important for me to focus on health issues that I am facing right now, not those that I might develop in the future” to evaluate present temporal orientation. This item had acceptable face validity with other instruments used to measure this cultural belief (14).

Participation in mammography screening was evaluated by self-report using items from previous studies (12, 15). First, women were asked if they had ever had a mammogram and those who replied yes were asked to report the month and year of their last mammogram. Since all interviews were completed during 2009, we used the self-reported year of the last mammogram to determine whether women were up-to-date on screening (i.e., having a mammogram in the preceding 12 months) using the ACS mammography guidelines in effect during the data collection period and recommending annual mammograms (4). Women who reported having a mammogram during 2008 or 2009 were categorized as being up-to-date. Women who reported not having a mammogram during 2008 or 2009 or never having a mammogram were categorized as being not up-to-date.

This study was approved and carried out in accordance with the recommendations of the Institutional Review Boards at the Medical University of South Carolina and the University of Pennsylvania.

### Statistical Methods

This descriptive study included a study analysis focused first on descriptive statistics to characterize respondents in terms of socioeconomics, health-care variables, and timely receipt of screening mammography. SAS version 9.3 was used to conduct multivariate logistic regression to identify factors having significant independent associations with mammography. To understand factors that might inform a woman’s decision to not have a mammogram we used multivariate logistic regression to examine the independent associations of socioeconomics, health-care variables, and psychological and culturally based preferences on the primary outcome of not having a mammogram within the last 12 months. Variables were included in the regression analysis based on factors showing significant associations with non-adherence and delays in seeking health-care services for breast care in previous studies that examined adherence to mammography guidelines (16–21). All statistical tests were two-sided with statistical significance set at the 0.05 alpha level.

## Results

Table 1 shows the characteristics of our study sample. Overall, 39% of women did not have a mammogram within the last 12 months as recommended by the ACS guidelines in effect during the study period. As shown in Table 2, our analysis of the influence of socioeconomic characteristics, health-care access, and cultural and psychological health-care preferences revealed several significant findings. Not having had a mammography screening varied significantly by racial group. The odds of not having been screened were higher for non-Hispanic White women than for non-Hispanic Black (OR = 2.16, 95% CI = 0.26, 0.82, *p* = 0.009) or Hispanic (OR = 4.17, 95% CI = 0.12, 0.48, *p* = 0.001) women. Conversely, the odds of not having been screened were lower for younger women (OR = 0.65, 95% CI = 0.48, 0.88, *p* = 0.01). The odds of not having been screened were higher for women without health insurance coverage (OR = 3.22, 95% CI = 1.54, 6.73, *p* = 0.002) and those without a usual source of medical care (OR = 3.37, 95% CI = 1.43, 7.94, *p* = 0.01). In addition, for our measure of self-efficacy, the odds of not having been screened were 2.43 times higher among women who were less confident about being able to obtain breast cancer screening (OR = 2.43, 95% CI = 1.26, 4.73, *p* = 0.01). Lastly, the odds of not having been screened were 1.42 times higher for women with greater levels of religious and spiritual values (OR = 1.42, 95% CI = 1.00, 2.00, *p* = 0.05). Neither perceived risk for developing breast cancer nor temporal orientation showed a significant association with not having had a mammography screening.

**Table 2:** **Logistic regression model of mammography non-adherence**.

Variable	Level	Odds ratio	95% confidence interval	*p*-Value
**Socioeconomic factors**				
Race	NH White vs. NH Black	2.16^b^	0.26^b^, 0.82^b^	*0.009*^b^
	NH White vs. Hispanic	4.17^b^	0.12^b^, 0.48^b^	*0.001*^b^
Marital status	Married	1.00	0.57, 1.77	0.99
	Not married			
Education level	>High school	0.90	0.50, 1.61	0.72
	≤High school			
Employment status	Employed	0.75	0.42, 1.34	0.33
	Not employed			
Income level	>$50,000	1.27	0.64, 2.50	0.49
	≤$50,000			
Age in years	^a^	*0.65*^b^	*0.48, 0.88*^b^	*0.01*^b^
**Health-care access factors**				
Health insurance	No	*3.22*^b^	*1.54*^b^, *6.73*^b^	*0.002*^b^
	Yes			
Usual source of medical care	No	*3.37*^b^	*1.43*^b^, *7.94*^b^	*0.01*^b^
	Yes			
**Psychological factors**				
Self-efficacy for screening	Not confident	2.43^b^	1.26^b^, 4.73	0.01^b^
	Confident			
Perceived risk for breast cancer	Not at risk	1.70	0.58, 4.99	0.33
	At risk			
**Cultural factors**				
Temporal orientation	Agree	0.70	0.39, 1.27	0.24
	Disagree			
Interpersonal relationships	^a^	0.98	0.72, 1.32	0.88
Religiosity	^a^	1.42^b^	1.00^b^, 2.00^b^	0.05^b^
Autonomy	^a^	0.88	0.68, 1.13	0.31

*p-Value means probability value, and NH means non-Hispanic*.

*Italic font indicates statistically significant findings*.

*^a^No level(s) reported*.

*^b^There was a significant association with non-adherence in the regression model*.

## Discussion

To our knowledge, our study is the first to evaluate the prevalence of not having a mammogram within the last 12 months based on socioeconomic characteristics, health-care access, and cultural and psychologically based preferences for cancer prevention and control in a national sample of racially diverse women. Consistent with other national studies (16, 22), about 40% of women in our study were not up-to-date with mammography guidelines at the time of the study.

Interestingly, non-Hispanic White women had significantly higher odds of not having been screened within the last 12 months as non-Hispanic Black and Hispanic women. This finding may be explained by several factors. First, these findings could relate to the extensive negative media attention about mammography in the US over the last decade, to the controversy among trusted and credible sources about initiation and timing of mammography screening, and to the relative pros and cons associated with new mammography technology such as 3D mammography. White women and women with higher educational and income levels may be more likely to be exposed to messages of harm outweighing benefit of mammography and to change their screening behavior as a result. Another possible reason for lower screening rates among White women and women with higher income levels may also be the extensive efforts undertaken to increase access to breast cancer screening among women from racial minority groups. The National Breast and Cervical Cancer Early Detection Program, for example, was established in 1990 to provide free and/or reduced cost mammograms to women with limited incomes and those who lack health insurance (23). Early reports from this program demonstrated that the percentage of women eligible for the program was smaller among non-Hispanic White women than among women from racial minority groups and Hispanic women had the highest screening rates (24). However, recent data show that percentages of White women screened through this program are higher than the percentages of African-American or Hispanic women screened. Specifically, of the women screened from July 2006 through June 2011, 43% were White, 16% were African-American, and 27% were Hispanic (25). Our finding that lower rates non-Hispanic Black and Hispanic women were not up-to-date on screening could be due to other free and/or reduced cost screening programs that specifically targeted women from racial and ethnic minority groups. Many community-based organizations offer free and/or low cost mammograms; non-Hispanic Black and Hispanic women may be using these resources to obtain mammograms. We did not ask women to indicate the type of facility where they obtained their last mammogram or how the cost for this service was paid. These variables should be measured in future studies about mammography use to determine how the distribution of and access to free and reduced cost screening contributes to racial differences in being up-to-date for breast cancer screening.

We also found women without health insurance coverage or a usual source of medical care had greater odds of not being up-to-date on screening. These findings are consistent with the results of other studies (16, 22) and underscore the necessity of health insurance and a usual source of medical care for obtaining preventive care. Recently, the Patient Protection and Affordable Care Act (ACA) was passed by the United States Congress to protect health-care consumers from discriminatory practices by insurance companies, provide better preventive care, and increase access to information that is necessary to make informed decisions about preventive services. This legislation is also designed to eliminate co-payments and deductibles for preventive care for women and provide education about disease prevention strategies. It will be important to determine if health insurance coverage and having a usual source of medical care have similar relationships with not being up-to-date for mammography screening as components of the (ACA) are implemented, especially given the considerable variability in how states meet federal requirements of this legislation.

Our findings show that while significant, health insurance coverage and having a usual source of medical care are not the only factors that influence participation in mammography screening. Women who were not confident in their ability to obtain breast cancer screening had about twice odds of being out-of-date for screening compared to those with greater levels of confidence. In addition, the odds of not being up-to-date increased with greater levels of religious and spiritual values for cancer prevention and control. Religious and spiritual beliefs are an important aspect of patient preferences for how health-care services are delivered (26–28) and influence patients’ decisions about seeking treatment for breast cancer symptoms (21). These factors are increasingly addressed as part of culturally tailored interventions for breast cancer screening (29–31). The premise of cultural tailoring is that information and messages customized to one’s culturally based beliefs and values will be more effective than generic approaches since they address issues that are most salient to an individual (28). However, the effects of culturally tailored approaches have been mixed (29) and, in some cases, have not been demonstrated to be more effective than non-tailored approaches for impacting health behaviors related to cancer prevention and control (31, 32).

Our findings offer some insight into why culturally tailored interventions designed to target-specific preferences related to breast cancer screening have had mixed results. Previous studies have shown that culturally based preferences and behaviors related to religion and spirituality have a positive association with screening and other preventive health care (28, 33). Our data show that this may not always be the case; the likelihood of being non-compliant increased with greater religious and spiritual values for cancer prevention and control. It could be that our findings differ from the results of other studies because our measure of religious and spiritual values assessed the extent to which individuals used spiritual and religious practices and relied on God or a higher power to protect their health. This practice may reduce the likelihood of performing behaviors designed to detect diseases early. But, religious and spiritual beliefs and values may be manifested in different ways; individuals with an internal religious or spiritual orientation may believe that they can influence their health through prayer whereas those with an external orientation may believe that others (e.g., physicians, pastors) have the power to impact their health and rely on these individuals for health advice and/or care (28). However, individuals who believe that their health is determined by God or a higher power may pray because they do not believe that there is anything they can do to influence their health (28). It should be noted that our measure is similar to the items used by Lannin et al. (21) to evaluate religious and spiritual beliefs in their study of cultural influences on treatment delays for breast cancer symptoms. Our measure is consistent with an external religious and spiritual orientation in which individuals relied on God or a higher power because this was how African-American, White, and Hispanic men and women described their religious and spiritual values for cancer prevention and control.

Study limitations include both temporal and study design issues. First, our data were collected as the recommendations for mammography were being modified by the USPSTF. Our data were collected prior to the release of the new recommendation in November 2009, but it could be that some screening decisions were affected by the expectation of change in the USPSTF recommendation. Since our measures of the prevalence of not being up-to-date on mammography screening are consistent with those reported in other national studies conducted while there was greater consistency in screening recommendations (22), it is likely that the time frame during which our data were collected did not have an impact on screening participation. Second, data on mammography use were obtained by self-report so it was not feasible to validate responses using medical records. While our study included women who were non-Hispanic Black, non-Hispanic White, and Hispanic and these are the three largest racial groups in the US, it is important to examine timely participation in mammography screening among women from other racial and ethnic backgrounds. Lastly, we had a modest response rate, likely due in part to overall declines in response rates for national surveys. For example, the Pew Research Center has shown that response rates for their telephone surveys declined from 36% in 1997 to 9% in 2012 (34). But, their recent study found that even with declining response rates, the samples enrolled in telephone surveys are likely to be similar to the US population in terms of demographics and other variables (34). As a measure to help control for the potential for sampling bias, we used population target estimates from the March 2009 Current Population Survey and weighted estimates for each racial group.

Despite these limitations, our findings reveal that health insurance and having a usual source of medical care are necessary, but not sufficient, factors for achieving timely participation in mammography screening. We found that women had higher odds of not being up-to-date on mammography screening if they were younger in age, not confident in their ability to obtain screening, and held passive or external religious and spiritual values for cancer prevention and control. Our findings also show that non-Hispanic White women had greater odds of not being screened within the last 12 months than non-Hispanic Black and Hispanic women. Relatively low rates of not being up-to-date with screening among minority women are encouraging in light of racial disparities in breast cancer outcomes. This finding suggests that the extensive efforts underway nationally to increase access to breast cancer screening among minority women have had the intended effect. But, our findings also suggest that an unintended consequence of these efforts may be lower rates of non-adherence among White women. Ultimately, all women should be able to access early detection strategies for breast cancer. Additional research is needed to ascertain how the distribution of and access to free and reduced cost screening as well as changes in socioeconomic inequalities may have been contributed to racial disparities in not being up-to-date for breast cancer screening, especially now that legislation has been passed that might mitigate these differences.

## Author Contributions

CM made substantial contributions to the analysis and interpretation of the data for the manuscript, prepared the initial and coordinated subsequent draft content of the manuscript, approved the final submitted version, and agreed to be accountable for all aspects of the manuscript. CHH had full access to all of the study data and took responsibility for the integrity of the data and the accuracy of the data analysis and results. CHH directed the initial survey work leading to this manuscript, conducted the analysis, had full access to all of the study data, reviewed and revised drafts of the manuscript, approved the final version submitted to the journal, took responsibility for the integrity of the data and the accuracy of the data analysis and results, and agreed to be accountable for all aspects of the manuscript. KC, MJ, and LR contributed substantially to the analysis and interpretation of data for the manuscript, assisted in revisions to the manuscript, approved the final submitted version, and agreed to be accountable for all aspects of the manuscript. All authors agreed to be accountable for their work.

## Conflict of Interest Statement

The authors declare that the research was conducted in the absence of any commercial or financial relationships that could be construed as a potential conflict of interest.
